# Kisspeptin and GPR54 Receptor Expression in Endometrial Cancer Tissue

**DOI:** 10.3390/cancers15041228

**Published:** 2023-02-15

**Authors:** Marek Gowkielewicz, Aleksandra Lipka, Aleksandra Piotrowska, Marta Szadurska-Noga, Jacek J. Nowakowski, Ewa Lepiarczyk, Marta Wiszpolska, Tomasz Waśniewski, Piotr Dzięgiel, Jerzy Kaleczyc, Mariusz Krzysztof Majewski, Marta Majewska

**Affiliations:** 1 Department of Gynecology and Obstetrics, School of Medicine, Collegium Medicum, University of Warmia and Mazury in Olsztyn, 10-045 Olsztyn, Poland; 2 Division of Histology and Embryology, Department of Human Morphology and Embryology, Wroclaw Medical University, Chałubińskiego Street 6a, 50-368 Wroclaw, Poland; 3 Department of Pathomorphology, School of Medicine, Collegium Medicum, University of Warmia and Mazury in Olsztyn, 10-561 Olsztyn, Poland; 4 Department of Ecology & Environmental Protection, University of Warmia and Mazury in Olsztyn, Plac Łódzki 3, 10-727 Olsztyn, Poland; 5 Department of Human Physiology, School of Medicine, Collegium Medicum, University of Warmia and Mazury in Olsztyn, 10-082 Olsztyn, Poland; 6 Division of Human Biology, Faculty of Physiotherapy, University School of Physical Education in Wroclaw, 51-612 Wroclaw, Poland; 7 Department of Animal Anatomy, Faculty of Veterinary Medicine, University of Warmia and Mazury, Oczapowskiego 13, 10-719 Olsztyn, Poland

**Keywords:** endometrial cancer, KISS, GPR54, AMH, AMHRII

## Abstract

**Simple Summary:**

Endometrial cancer (EC) is the most common malignant neoplasm of the female genitalia. Depending on the type and stage of EC, different classical treatment methods are used. Those methods could be supported by targeted medicine—a form of cancer therapy based on the influence of certain substances on specific cellular mechanisms, resulting in the inhibition of cell division in the tumor. The basic condition is the presence of receptors for those agents in the malignant cells. Kisspeptin (KISS) is a natural peptide with properties inhibiting the growth of metastatic cells; moreover, it induces apoptosis in them. KISS has also a predictive value—its lower expression correlates with a poor prognosis. KISS has a favorable toxicity profile—it does not cause significant side effects. The study aims to analyze the expression of KISS and its receptor in cells of various histological types of the EC in correlation with concomitant diseases and biometric characteristics.

**Abstract:**

Kisspeptin (KISS) is a natural peptide—discovered in 1996 as a factor inhibiting the ability to metastasize in malignant melanoma. This protein plays also a regulatory role in the process of puberty, the menstrual cycle, spermatogenesis, implantation and development of the human placenta. The present study aimed to evaluate the expression of KISS and its receptor GPR54 in endometrial cancer (EC) tissue, depending on the histological type of cancer, its stage, various demographic characteristics, and clinical conditions in 214 hysterectomy patients. Expression of KISS and GPR54 was confirmed in 99.5% and 100% of the cases, respectively. Hormone replacement therapy and the coexistence of the anti-Müllerian type 2 receptor in cancer tissue enhanced KISS expression. Smoking, on the other hand, decreased KISS expression. GPR54 expression increased with the advancement of the disease (according to FIGO classification). Also, the presence of the anti-Müllerian type 2 receptor in EC increased the level of GPR54. Hypertension, age and miscarriage harmed the presence of GPR54. The histological type of cancer, diabetes type 2, body mass index, hormonal contraception, number of deliveries, birth weight of newborns, breastfeeding time, and the presence of AMH in EC tissue were not associated with the expression of either KISS nor GPR54. The KISS level was also significantly related to the GPR54 level. Considering that KISS is a non-toxic peptide with antimetastatic properties, further investigation is essential to determine the clinical significance of this peptide.

## 1. Introduction

Kisspeptin (KISS) was first described in 1996 as a tumor metastasis suppressor in melanoma cells [1]. Further studies revealed that KISS is a neuropeptide involved in the hypothalamic-pituitary-gonadal (HPG) axis, controlling all stages of reproduction. KISS is required for puberty onset and maintenance of reproductive functions; it acts as a key upstream regulator of GnRH and mediates sex steroid feedback and metabolic input in the reproductive axis [2]. As KISS is a factor involved in the HPG axis, its analogues have recently been taken into account in the treatment of endocrinological disorders as polycystic ovarian syndrome and hypothalamic amenorrhea. Also, they are considered as agents helpful in the management of endometriosis and in the protocols of in vitro fertilization as protection against ovarian hyperstimulation syndrome [3,4,5]. The product of the *KISS1* gene is a peptide of 54 amino acids; however, this can be processed into smaller peptides, including kisspeptin-10 (Kp-10), Kp-13, and Kp-14, sharing similar functions and activities [6]. KISS functions via the GPR54 receptor (also known as KISS1R, AXOR12, CPPB1, hOT7T175 or HH8). GPR54 consists of seven transmembrane domains and belongs to the family of G protein-coupled receptors [7,8]. Outside the hypothalamic area, the distribution of GPR54 is consistent with KISS—both are highly expressed in placental tissue, central nervous system, pancreas, and liver, which indicates the association of KISS with metabolism and homeostasis [1,9,10].

KISS has been reported to suppress the metastatic capabilities of various types of cancer cells, through the activation of its GPR54 receptor [11]. The KISS/GPR54 complex as a negative regulator of cancer metastasis was confirmed in breast, gastric, pancreas, bladder and prostate cancer [1,7,10]. The metastasis inhibitory potential is achieved by restraining cell motility, proliferation, invasion, and chemotaxis [12]. The specific mechanisms through which KISS suppresses tumor metastasis are not yet established. However, a few signal transduction pathways, such as calcium mobilization through Gq activation and regulation of matrix metalloproteinases, are indicated as key downstream events of the KISS/GPR54 complex [13,14].

The expression of the KISS/GPR54 system in the endometrium varies with the phases of the menstrual cycle. During the proliferative and early secretory phases, it was detected in epithelial cells, but not in stromal cells [15]. Contrariwise, in the late secretory phase, KISS is highly expressed in the endometrial stromal cells, potentially regulating decidualization in preparation for adequate placentation [8,15]. In women suffering from endometriosis, it was shown that KISS expression was significantly increased in the ectopic glandular endometrium, compared with patients without endometriosis [16]. However, no differences were found in the expression of KISS in stromal cells from patients with endometriosis, compared with healthy patients. Thus, it is suggested that KISS may be potentially involved in the pathogenesis and maintenance of endometriosis [8,16].

Ovarian cancer is one of the leading cancers in women, causing more deaths than any other cancer in the female reproductive system [17]. Patients with increased expression of KISS and GPR54 receptors have a better prognosis, as increased expression of this system reduces metastasis and inhibits cell migration [9,17]. Moreover, the lower the KISS/GPR54 system expression, the worse the prognosis in epithelial ovarian cancers [9,17,18]. It is believed that the *KISS1*/*GPR54* complex expression level can be considered a biomarker of disease progression in ovarian cancer [17].

Another major cause of malignant gynecological disease is endometrial cancer (EC), and the extrauterine spread of this cancer is a factor with a huge impact on patient prognosis [19]. Thus, the inhibition of the metastatic capability is an important issue for improving the prognosis of women diagnosed with EC. It was suggested that kisspeptin-10 in combination with demethylating agents inducing *GPR54* expression, may be effective in reducing the spread of endometrial cancer metastasis. Also, *GPR54* expression is associated with well-known prognostic factors, including FIGO stage, grade, and deep myometrial invasion [19]. Our previous studies regarding anti-Müllerian hormone (AMH) and anti-Müllerian hormone receptor type 2 (AMHRII) expression in EC revealed that these molecules can be considered as potential new indicators in oncology to manage advanced, recurrent, and metastatic EC [20,21]. The current research aimed to determine KISS and GPR54 expression levels in tissues of various types of EC, in terms of comorbidities, patient characteristics, tumor malignancy, stage, histological type, and grade, as well AMH and AMHRII expression.

## 2. Materials and Methods

### 2.1. Samples

As previously described [20,21], the study was performed on archived paraffin blocks of EC obtained from 214 patients who underwent surgical removal of precancerous or cancerous endometrial lesions (hysterectomy with bilateral salpingo-oophorectomy, in most cases accompanied by lymphadenectomy, as well as peritoneal washing in the Clinical Ward of Gynecology, Obstetrics and Oncological Gynecology at the Regional Specialist Hospital in Olsztyn, Poland). The study was approved by the Bioethics Committee of the Warmia-Mazury Medical Chamber (OIL. 164/15/Bioet; 2 April 2015) in Olsztyn, Poland. Immediately after surgical removal, tissue samples were preserved in 4% buffered formaldehyde (ready-to-use phosphate-buffered solution, Chempur, Poland), and the volume of fixative-to-tissue ratio was at least 10:1. All samples received a minimum of 6 h and a maximum of 12 h fixation at room temperature (22–25 °C). The preserved tissues were placed in cassettes and processed using an automated routine (Leica ASP 300S, Leica Biosystems, Nussloch, Germany) paraffin embedding station (Leica EG1160). For all patients, case history reviews including demographic details, tumor characteristics, comorbidities and survival outcomes were collected.

### 2.2. Immunohistochemistry

Paraffin blocks were cut into sections (6 mm) that were stained with hematoxylin and eosin (H&E) and evaluated by an experienced pathologist. Then, slides were scanned using the Pannoramic MIDI II histological scanner (3DHistech; Budapest, Hungary). PannoramicViewer (3DHistech) software was used to manually select three representative areas of a surface of 1.5 mm^2^, from regions of EC previously indicated by the pathologist. Three representative cores of 1.5-mm diameter were cut from each sample of an EC tumor and embedded in paraffin to create tissue microarrays (TMAs) using TMAGrand Master (3DHistech), in line with the manufacturer’s instructions. TMA slides (4 mm thick) were used for the detection of KISS1 and GPR54 with immunohistochemistry (IHC) reactions, performed using an automated system (DakoAutostainer Link48, Dako, Glostrup, Denmark). Briefly, using the PTLink platform (Dako, Glostrup, Denmark), slides were deparaffinized, rehydrated and boiled in EnVision FLEX Target Retrieval Solution (pH 9, 20 min, 97 °C; Dako) to unmask the antigens. Afterwards, to block endogenous peroxidase, slides were incubated for 5 min with Envision Flex Peroxidase-Blocking Reagent (Dako, Glostrup, Denmark). As primary antibodies (20 min, RT), rabbit polyclonal antibodies against KISS1 (1:200, orb10955, Biorbyt Ltd., Cambridge, UK) and GPR54 (1:100, orb522544, Biorbyt Ltd., Cambridge, UK) were used. Then, slides were incubated with EnVision FLEX/HRP (20 min, RT), and the reaction was visualized (10 min, RT) with freshly prepared 3,30-diaminobenzidine (DAB). To visualize the samples’ morphology, slides were stained for 5 min with EnVision FLEX Hematoxylin (Dako, Glostrup, Denmark). Finally, slides were dehydrated in ethanol (70%, 96%, absolute) and xylene, and then mounted with Dako Mounting Medium (Dako, Glostrup, Denmark). Expression of the KISS1 and GPR54 was evaluated using the Olympus BX41 light microscope (Olympus, Japan). Human lung cancer tissue was used as a control specimen. The performed immunohistochemical reactions were evaluated by two pathologists using a BX-41 light microscope, and for evaluation, an immunoreactive score of Remmele and Stegner was applied [22].

### 2.3. Statistical Analysis

KISS1 protein and GPR54 receptor expression were scored with the semi-quantitative rank scale ISR (point scale: 0–12) in three samples retrieved from three randomly collected places of cancer tissue from each patient. The mean or median value of the three measurements for each patient was computed and used to analyze the differential expression of the KISS1 protein and its receptor.

The dependence of KISS protein/GPR54 expression on other factors (age of women, BMI, number of miscarriages, number of deliveries, newborn mass, breastfeeding time, hypertension, smoking of classic cigarettes), in the first steps, were tested in generalized linear and nonlinear models (GLZ) with the logarithmic link function (f(z) = log(z)). The maximum likelihood (ML) method was used to build models and estimate the parameters [23]. The selection of the best-fitting model was made based on the Akaike information criterion [24]. Unfortunately, all analyses for the KISS protein reduced the model to a single significantly associated factor, HRT. No interaction between factors was found. In the case of GPR54, no satisfactory model was fitted. Therefore, the results of the one-dimensional analyses are presented.

To choose an appropriate approach for further statistical analysis, all samples were tested for compliance with the normal distribution using the Shapiro–Wilk test [25]. Differences in expression between tumor types and tumor developmental types according to the FIGO scale were tested with the Kruskal-Wallis test or median test for equalities, and differences between the two groups of women (women with diagnosed hypertension and normal blood pressure; hormonal contraceptive users and nonusers; women using hormone replacement therapy and not; women with and without diabetes; and smokers and nonsmokers) were tested using Mann-Whitney test [26].

The association of the KISS protein and GPR54 expression level with quantitative variables (age, BMI, number of births, number of miscarriages, mean newborn weight, mean breastfeeding time, AMH, AMHRII level) was tested using the Spearman correlation coefficient, or, due to multiple linking of the same ranks, a Goodman and Kruskal’s gamma correlation coefficient [26,27]. The polynomial equation as the best-fitted model was used to describe the relationship between the KISS protein and receptor expression. All analyses were performed using Statistica 13.3 (TIBCO, 2017) and SPSS Statistics 28 (IBM 2022). Statistical significance was set at *p* < 0.05.

## 3. Results

**Figure 1:**
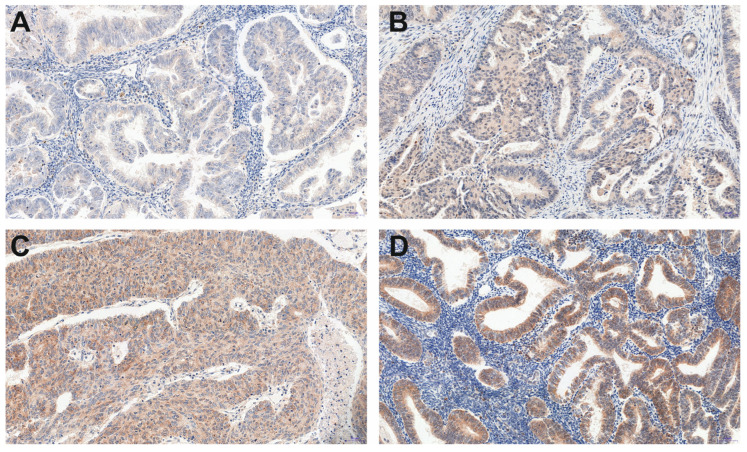
A representative example (200× magnification) of the weak (**A**), moderate (**B**), and strong (**C**,**D**) KISS immunohistochemistry (IHC) reaction within endometrioid adenocarcinoma G1 (**D**), G2 (**A**,**B**) and G3 (**C**) grade.

**Figure 2:**
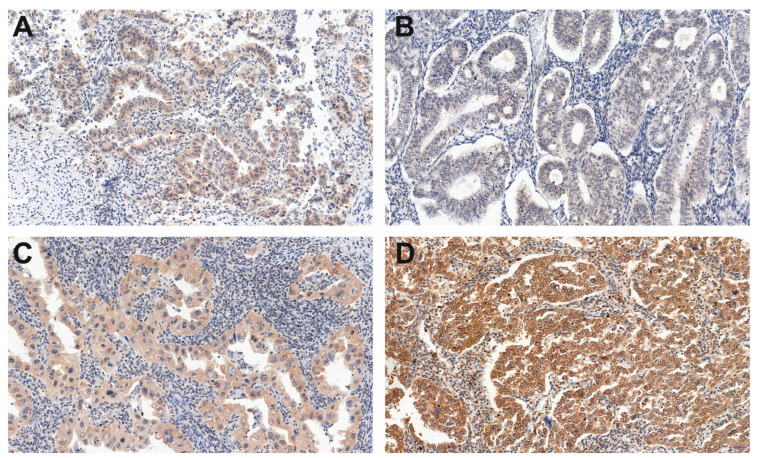
A representative example (200× magnification) of the weak (**A**), moderate (**B**,**C**) and strong (**D**) GPR54 immunohistochemistry (IHC) reaction within endometrioid adenocarcinoma G1 (**B**), G2 (**C**,**D**) and G3 (**A**) grade.

Among 214 tissue microarray (TMA) specimens, all showed a positive KISS and GPR54 reaction (Figure 1 and Figure 2).

The detected expression and its mean values are presented in Table 1.

**Figure 3:**
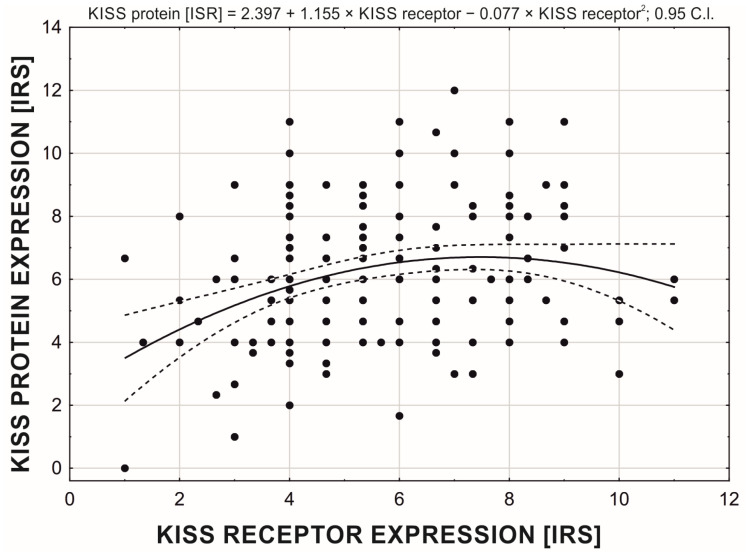
Relationship between expression of GPR54 and KISS protein.

Overall, KISS protein and GPR54 receptor expression levels were positively associated, with the relationship arranged according to a second-order polynomial function (KISS protein [ISR] = 2.397 + 1.155 × KISS receptor − 0.077 × KISS receptor^2^). This indicates that, up to an ISR level 7–8 of the receptor, protein expression increased (ISR 6–7), while at higher receptor expression, protein expression decreased (Figure 3). The protein expression was significantly higher (ISR: Me = 6.0) than receptor expression (ISR: Me = 5.3) in the studied group of women (Wilcoxon test: T = 7173.0; Z-2.289; *p* = 0.022).

**Figure 4:**
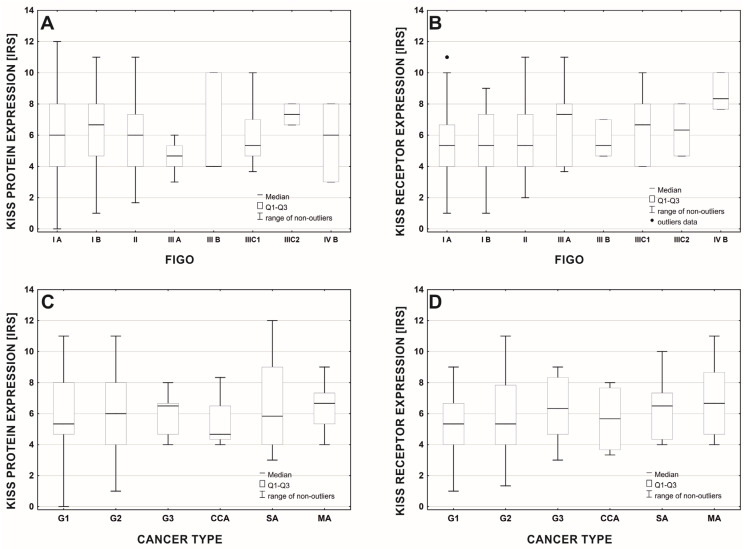
Mean expression of the KISS protein (**A**,**C**) and GPR54 (**B**,**D**) in different stages of cancer according to FIGO (**A**,**B**) and histopathological types of cancer (**C**,**D**). Abbreviations: G1—endometrioid adenocarcinoma G1; G2—endometrioid adenocarcinoma G2; G3—endometrioid adenocarcinoma G3; SA—serous adenocarcinoma; CCA—clear cell adenocarcinoma; MA—mixed adenocarcinoma.

There were no significant changes in KISS protein expression (Figure 4A) between stages of tumor development, according to FIGO classification (median test: chisq = 10.634, df = 7, *p* = 0.155; rgamma = −0.065; *n* = 214; *p* = 0.261), but we found that GPR54 expression increased with the stage of tumor advancement (Figure 4B) (i.e., the difference between developmental stages was significant at the accepted level of probability—median test: chisq = 20.965, df = 7, *p* = 0.004). The difference is significant for pairwise comparisons of receptor expression in tumor stages IA, IB, and II, relative to stage IVB (test Jonkheere-Tapestra: *p*-0.010–0.025), but not at the level of comparison for error estimated across all stages (Bonferroni correction: *p* > 0.05). The rank correlation coefficient for the relationship between the ranks of tumor stages according to FIGO classification and the level of GPR54 expression indicates a significant positive relationship (rgamma = 0.144; *n* = 214, *p* = 0.013). There was no significant difference in KISS protein (Figure 4C) and GPR54 expression (Figure 4D) levels between tumor histopathological types (Kruskal-Wallis test; protein: H(5, N = 214) = 0.752; *p* = 0.980; receptor H(5, N = 214) = 3.366; *p* = 0.644).

**Figure 5:**
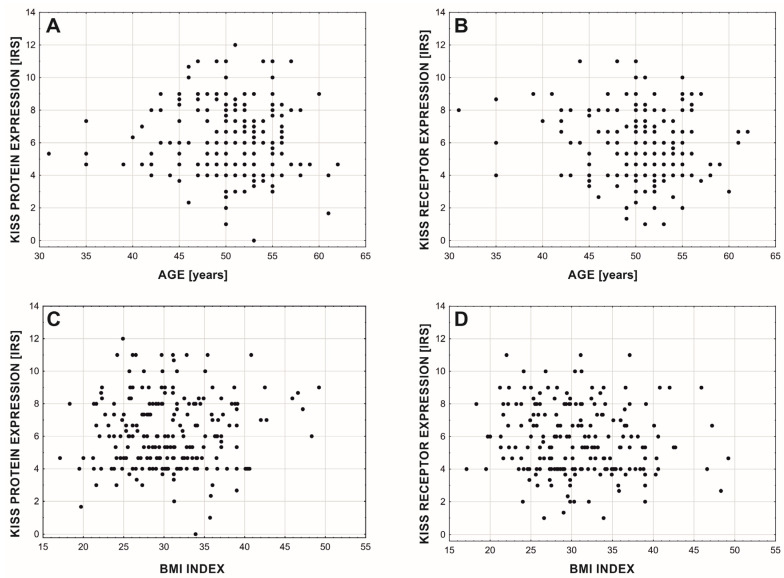
Relationship between age (**A**,**B**) and BMI (**C**,**D**) of patients and expression of the KISS protein (**A**,**C**) and GPR54 (**B**,**D**).

As the patient aged, the protein expression levels did not change (Figure 5A; rgamma = −0.048; *p* = 0.343; *n* = 214), while there was a statistically significant but very slight decrease in GPR54 expression (Figure 5B; rgamma = −0.100; *p* = 0.048; *n* = 214). Also, we did not find any significant changes in the expression of the protein and its receptor affected by BMI (Figure 5C,D depicting protein (rs = 0.012; *p* = 0.861; *n* = 214) and receptor (rs = −0.131; *p* = 0.056; *n* = 214) analyses).

**Figure 6:**
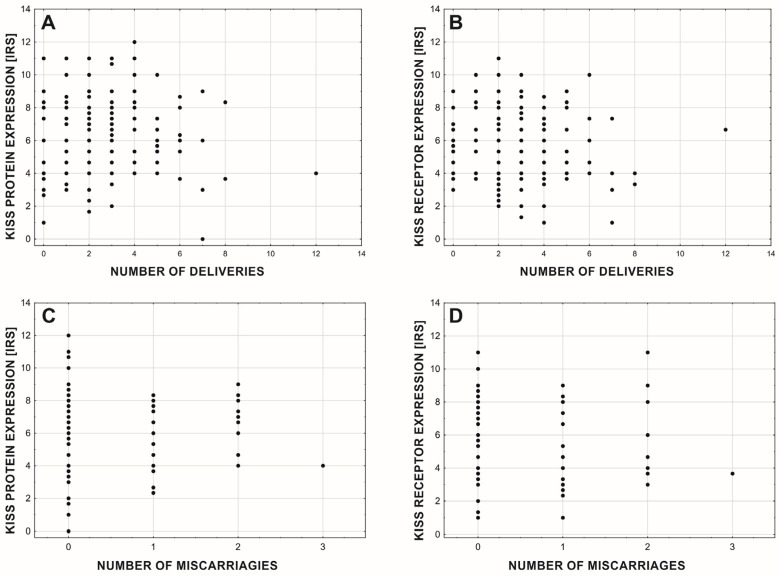
Mean expression of KISS protein (**A**,**C**) and GPR54 (**B**,**D**) due to the number of births (**A**,**B**) and miscarriages (**C**,**D**).

The expression levels of the KISS protein and its receptor were not affected by the number of births (Protein: rgamma = 0.031; *p* = 0.569; *n* = 214; Figure 6A. Receptor: rgamma = −0.077; *p* = 0.153; *n* = 214; Figure 6B) but the level of GPR54 expression slightly (but significantly) decreased with the numbers of the miscarriages (Protein: rgamma = −0.185; *p* = 0.028; *n* = 214; Figure 6C. Receptor: rgamma = −0.212; *p* = 0.010; *n* = 214; Figure 6D).

**Figure 7:**
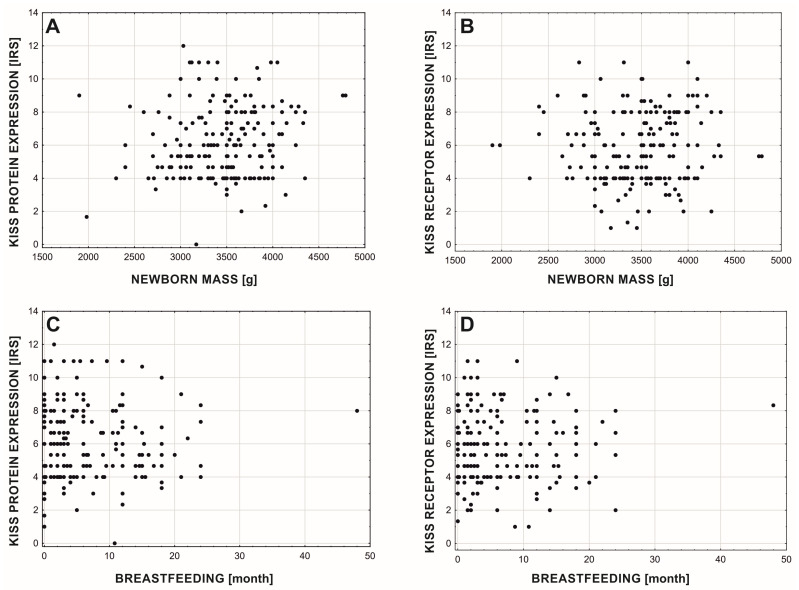
Relationship between average newborn weight (**A**,**B**) and average duration of breastfeeding and expression of KISS protein (**A**,**C**) and GPR54 (**B**,**D**).

There were no significant differences in the protein and its receptor expression between women with and without diabetes type 2 (Mann-Whitney test for protein (U = 4036.5; Z = 0.310; *p* = 0.757; nno = 163, nyes = 51) and receptor (U = 3850.5; Z = 0.792; *p* = 0.429; nno = 163, nyes = 51)). Also, we did not find any significant changes in the expression of protein and its receptor affected by average newborn weight (Figure 7A,B depicting protein (rs = 0.114; *p* = 0.115; *n* = 192) and receptor (rs = 0.048; *p* = 0.509; *n* = 192) expression) and average duration of breastfeeding (Figure 7C,D depicting protein (rs = −0.024; *p* = 0.717; *n* = 214) and receptor (rs = −0.017; *p* = 0.805; *n* = 214) expression).

**Figure 8:**
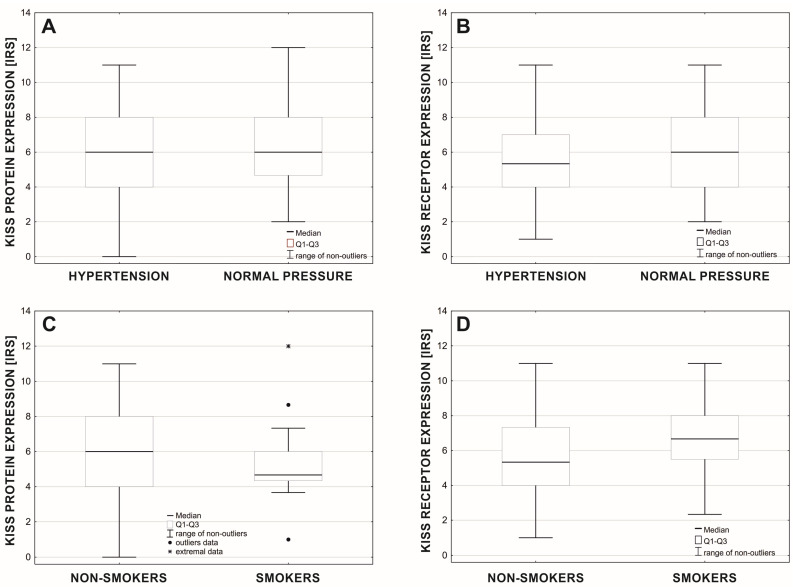
Expression of the KISS protein (**A**,**C**) and GPR54 (**B**,**D**) connected to diagnosed hypertension and normal blood pressure (**A**,**B**), and regarding the group of smokers and non-smokers (**C**,**D**).

The differential expression of the KISS protein and receptor was observed between groups of women with diagnosed hypertension and normal blood pressure (Figure 8). The expression of the KISS protein did not differ between (Figure 8A) these two groups (Mann-Whitney test: U = 5376.5; Z = 0.135; *p* = 0.893; nno = 83, nyes = 131), but the level of GPR54 was significantly higher (Figure 8B) in women with normal blood pressure (Me = 6.0) compared with the group with diagnosed hypertension (Me = 5.3) (Mann-Whitney test: U = 4464.0; Z = −2.202; *p* = 0.028; nno = 83, nyes = 131). The response of protein and receptor expression also differed between smokers and non-smokers (Figure 8). Protein expression was significantly higher (Figure 8C) in the non-smoking group (Mann-Whitney test: U = 1698.0; Z = 2.034; *p* = 0.042; nno = 190, nyes = 24), while receptor expression was higher (Figure 8D) in the smoking group (Mann-Whitney test: U = 1673.5; Z = −2.120; *p* = 0.034; nno = 190, nyes = 24).

**Figure 9:**
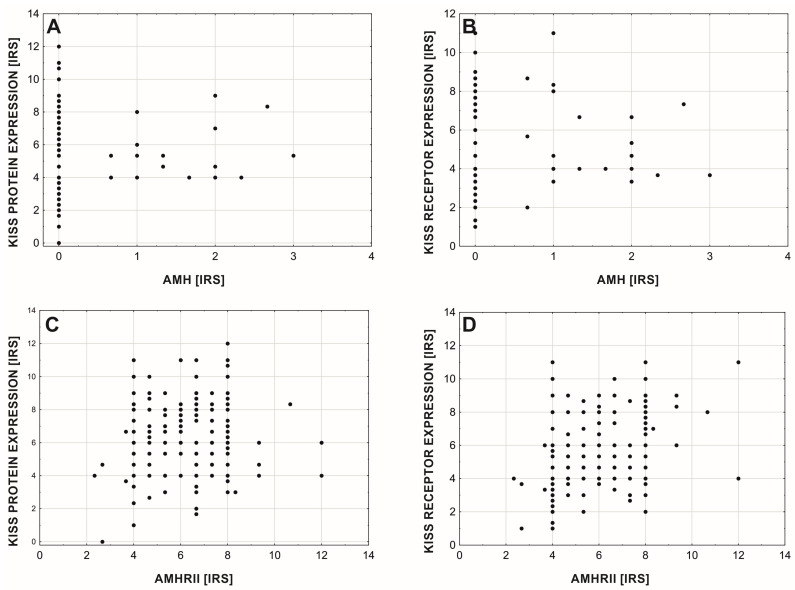
Expression of KISS protein (**A**,**C**) and GPR54 (**B**,**D**) with regard to AMH (**A**,**B**) and AMHRII expression (**C**,**D**).

There were no significant correlations between AMH protein expression and KISS protein (Figure 9A; rgamma = −0.223, *p* = 0.052, *n* = 214) and its receptor (Figure 9B; rgamma = −0.153, *p* = 0. 165, *n* = 214), but the AMHRII expression level was significantly positively correlated with KISS protein expression level (Figure 9C; rs = 0.202, *p* = 0.003, *n* = 214) and GPR54 expression level (Figure 9D; rs = 0.277, *p* = 0.00004, *n* = 214).

**Figure 10:**
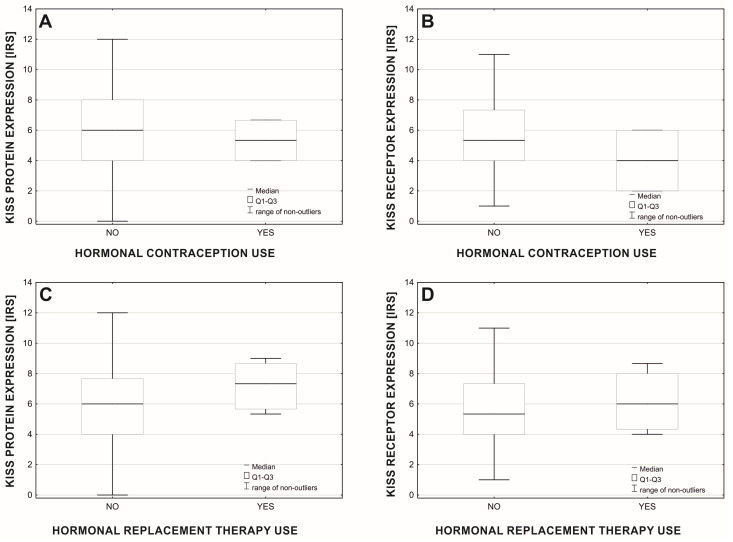
Mean expression of KISS protein (**A**,**C**) and GPR54 (**B**,**D**) in terms of use of hormonal contraception (**A**,**B**) and hormonal replacement therapy (**C**,**D**).

Analysis of the differential expression of the studied protein and its receptor concerning the use of hormonal contraception or hormone replacement therapy (HRT; Figure 10), revealed that the expression of the KISS protein did not differ between women using hormonal contraception and women not using hormonal contraception (Mann-Whitney test: U = 161.5; *p* = 0.578; nno = 210, nyes = 2). However, the expression of KISS was higher (Figure 10C) in the group of women using HRT (ISR: 7.2 vs. 6.0) (Mann-Whitney test: U = 729.5; *p* = 0.025; nno = 197, nyes = 12), compared with non-users. There was no significant difference in GPR54 expression between users and non-users of hormonal contraception (Mann-Whitney test: U = 126.0; *p* = 0.334; nno = 210, nyes = 2), and users of HRT in comparison to non-users (Mann-Whitney test: U = 1039.5; *p* = 0.485; nno = 197, nyes = 12).

## 4. Discussion

In the current research, we analyzed a cohort of EC samples to determine KISS and GPR54 expression levels in terms of various patient features and tumor characteristics, including malignancy, stage and histological type. This study is a continuation of our previous investigations focused on AMH and AMHRII expressions in endometrial cancer [20,21]. The main idea of the undertaken research was to provide insight into the expression of antimetastatic agents such as KISS and AMH, and to determine their potential utility as targets for new oncological therapies.

The antimetastatic effect of KISS, as well as the lack of its toxicity, were confirmed in many studies; therefore, this peptide has become the subject of numerous discussions as a component of potential oncological therapy [19,28,29,30,31]. Decreased KISS expression is associated with worsened prognosis and a greater chance of metastasis in ovarian, breast, bladder, gastric and esophageal cancer [18,32,33,34,35]. However, there is no differentiation of KISS expression between histopathological types of tumors and the expression itself does not affect the overall survival of patients with endometrial cancer [19]. Our results remain in line with available data, as no variation in KISS expression was found between tumor staging, according to FIGO classification, and also between tumor histopathological types. Current research also revealed that some other factors, such as age, BMI, parity, number of miscarriages, the average time of breastfeeding, average birth weight of newborns and the use of hormonal contraception, and comorbid diseases such as diabetes mellitus type 2 were irrelevant in this context.

The obtained results revealed that KISS expression in EC tissue is associated with the level of GPR54; this fact reflects about 5% of protein variability. Specifically, the expression of the GPR54 receptor positively influences the expression of KISS up to a certain point, and then, with increasing levels of GPR54, the tissue representation of the KISS decreases. Our results also showed an increase in GPR54 expression, with values higher than the median in more advanced stages of cancer according to the FIGO classification, which, with correlation to lower levels of KISS, could explain a worse prognosis in these cases. Obtained outcomes are consistent with the literature data concerning KISS variability, where the aggressiveness of cancer and the advancement of neoplastic disease both increase with the decrease in KISS expression [36,37,38]. However, it should be mentioned that other research has presented contradictory results concerning the expression of GPR54 in EC tissue, with a decreased level of the receptor in conjunction with age, FIGO stage, histopathological grade, myometrial invasion, and lymph node metastasis [19].

KISS expression, synthesis and release are directly linked to the current metabolic status of the organism. KISS is considered a transmitter of real-time information on circulating nutrients and stored energetic reserves [39]. Increasing body mass in childhood is a cause of activation of the HPG axis by kisspeptin and subsequently, premature puberty [40,41]. As BMI may also reflect the metabolic state of the organism, changes in KISS expression in terms of this index were suspected. However, we found no relationship between BMI and the expression of either kisspeptin or its receptor in EC tissue.

Available data regarding KISS correlation with birthweight are inconsistent [42]. Some research indicates that plasma kisspeptin levels during the first trimester were significantly lower in pregnancies of obese women and pregnancies with small-for-gestational-age neonates, and that kisspeptin levels in the second trimester (at 16th–20th week) in maternal plasma were positively associated with birthweight [43,44,45]. On the other hand, among two groups of patients, pregnancy with preeclampsia and uncomplicated pregnancy, no significant correlation was observed between plasma KISS levels throughout pregnancy and birth weight at delivery [46]. In the current research, the birthweight of the offspring did not affect the variability of kisspeptin and GPR54 in EC tissue.

It was also suggested that the KISS plasma level is significantly lower in pregnancies affected by miscarriage than in uncomplicated pregnancies [47]. There is a reliable connection between miscarriages and the KISS/GPR54 system in the literature on the subject. Recurrent pregnancy loss reduces KISS expression in decidua and trophoblast and does not alter GPR54 expression [41,48]. It has been proven, however, that GPR54 is lower in trophoblasts in the group of women with recurrent spontaneous abortion, while it remains unchanged in the decidua [41]. The performed analysis revealed that the increasing number of miscarriages slightly negatively affected the expression of GPR54 in EC tissue but had no influence on KISS expression.

In the period of the lactation experience, studies indicate the possibility that the suckling stimulus may suppress the kisspeptin peptide release and/or its axonal transport [49,50]. However, the length of lactations did not affect KISS expression and its receptor in EC tissue.

Currently, detected higher expression of GPR54 in EC tissue in patients with normal arterial pressure, compared with the group suffering from hypertension, is a very interesting result. Previous reports indicate that decreased plasma KISS levels in each trimester of pregnancy may be considered a biomarker of preeclampsia [43,44,51]. On the other hand, high placental expression of KISS also heralds preeclampsia [51]. Additionally, the literature on the subject provides data that kisspeptin-10 is not only a factor limiting trophoblast invasion but is also a vasoconstrictor, an angiogenesis inhibitor, and a factor accelerating the formation of plaques in atherosclerosis and its instability [8,51,52,53]. However, hypertension artificially elicited by angiotensin II does not change the mRNA level of GPR54 in the heart, aorta and kidney [54]. The obtained data are valuable in the cognitive context, but their interpretation is difficult, since the issue of hypertension, neurokinin B and KISS are intermingled in the available publications. The factor stimulating KISS release from the arcuate nucleus of the hypothalamus is neurokinin B [55,56,57,58]. Interestingly, this neuropeptide has been found at significantly higher concentrations in the plasma of people suffering from non-dipper hypertension (which carries a much higher risk of cardiovascular complications), compared with patients with dipper hypertension [59]. Certainly, the association between the KISS/GPR54 system and hypertension should be further investigated.

Various studies show data regarding the role of KISS at each stage of the reproductive cycle [39,60]. A subpopulation of KISS-positive neurons stays in close contact with GnRH-secreting neurons, determining KISS and its physiological role as a GnRH release modulator [61]. In the hormonal context, an interesting conclusion arises from the fact that HRT significantly increased levels of KISS in EC tissue in the analysed cohort. Previous studies highlighted HRT as a factor for differential response to KISS infusion [62]. Postmenopausal women not using HRT do not respond with an increased LH secretion to continuous KISS infusion but postmenopausal women using HRT respond with pulsatile LH secretion adequately to plasma estrogen concentration and infusion time [62]. Moreover, postmenopausal women show higher gonadotropin responses to KISS than follicular phase women, whereas healthy women on combined oral contraceptive pills show diminished responsiveness to exogenous KISS [63]. Although the reasons for different responsiveness to KISS administration are not clear, it is hypothesized that the ambient level of endogenous kisspeptin may be higher in the follicular phase than in other phases of the ovarian cycle and that this determines the impact of the exogenous KISS effect [64]. Responsiveness to KISS administration and KISS expression in EC tissue should be considered separately; however, those dependencies may suggest that the significant effect of HRT and no effect of hormonal contraception on KISS expression in EC tissue demonstrated in this study, may be caused by general hormonal status. Thus, taking into account KISS as a part of potential oncological treatment, all aspects of KISS expression should be assessed, including KISS as an essential element of neuroendocrine mechanisms responsible for GnRH-induced gonadotropin secretion, in both normal and pathophysiological states [65,66].

It is also postulated that sex steroid feedback occurs in the hypothalamus, as well as the pituitary gland, and this could explain different KISS effects in menstruating and post-menopausal women [63]. Studies performed in animal models showed that the age does not change KISS expression, but it affects GPR54 expression. Expression, decreasing with age, of GPR54 was also noted in the rat brain (hypothalamus, hippocampus, medulla and pons) [67]. On the other hand, the expression of GPR54 mRNA increased with age in human and mouse ovaries [68]. Therefore, we expected that factors such as age, parity, and hormonal contraception would directly translate into KISS/GPR54 expression as a consequence of long-term modulation and changes in HPG axis functioning. However, only GPR54 expression slightly decreased with age in EC tissue. The mentioned studies and current results suggest that age impact on KISS/GPR54 expression may be tissue-dependent and should not be correlated with the plasma level of KISS protein.

Smoking cigarettes significantly reduces the KISS expression in EC tissue. The effect of smoking on KISS expression is a very interesting issue. It was revealed that male smokeless tobacco users have increased levels of plasma KISS, compared with tobacco smokers or men not using any form of tobacco [69]. This phenomenon is probably related to the central effect of smokeless tobacco [69]. Among patients analyzed in the current study, none of the women used smokeless tobacco, so it makes it impossible to speculate about the effect of different tobacco forms on KISS expression in EC. Nevertheless, taking under consideration the increasing popularity of e-cigarettes and other alternative tobacco forms, it would be important to assess how it may affect KISS levels.

## 5. Conclusions

Tissue representation of AMH in the EC, although positively dependent on the histopathological type of cancer, the long average time of breastfeeding and 40 or more years of hormonal activity [20] does not correlate with the expression of KISS and its receptor. Both natural peptides are considered anti-cancer agents; however, the above fact suggests their different expression patterns in EC. There is a positive, but weak, relationship between KISS and AMHRII expression and between GPR54 and AMHRII in EC. It is worth mentioning that none of the factors (e.g., histopathological type of EC, FIGO stage, age, BMI, parity, time of breastfeeding, etc.) has an influence on the expression of AMHRII in EC, except the presence of diabetes mellitus type 2, which decreases this expression [21].

In a modern approach to cancer therapy, understanding the biology of neoplasms and the relationships between the expression of anti-cancer agents and their receptors in neoplastic tissue opens the way to the theoretical application of natural peptides, usually characterized by a safe toxicity index.

## Figures and Tables

**Table 1:** Summary of KISS protein and GPR54 expression in different histopathological types of endometrial lesions, with number of patients in each group, mean ± Standard Deviation and minimal and maximal values of expression.

Histopathological Type of Endometrial Lesion	Number of Patients	Variable	Mean ± SD	Minimal Expression	Maximal Expression
Endometrioid adenocarcinoma G1	47	KISS	5.96 ± 2.28	0.00	11.00
		GPR54	5.35 ± 1.97	1.00	9.00
Endometrioid adenocarcinoma G2	144	KISS	6.20 ± 2.17	1.00	11.00
		GPR54	5.76 ± 2.08	1.33	11.00
Endometrioid adenocarcinoma G3	6	KISS	6.06 ± 1.47	4.00	8.00
		GPR54	6.28 ± 2.32	3.00	9.00
Serous adenocarcinoma (SA)	8	KISS	6.58 ± 3.31	3.00	12.00
		GPR54	6.29 ± 2.06	4.00	10.00
Clear cell adenocarcinoma (CCA)	4	KISS	5.42 ± 1.97	4.00	8.00
		GPR54	5.67 ± 2.34	3.33	8.00
Mixed adenocarcinoma (MA)	5	KISS	6.47 ± 1.91	4.00	9.00
		GPR54	7.00 ± 2.89	4.00	11.00

## Data Availability

The data presented in this study are available in this article.
